# A decision support system for fault detection and definition of the quality of wet blue goat skins

**DOI:** 10.1016/j.heliyon.2021.e08021

**Published:** 2021-09-21

**Authors:** Carlos E.B. Sousa, Cláudio M.S. Medeiros, Renato F. Pereira, Alcides A. Neto, Mateus A.V. Neto

**Affiliations:** Federal Institute of Education, Science and Technology of Ceará - Fortaleza - CE, Brazil

**Keywords:** Fault detection in leather, Qualification of leather parts, Decision support system

## Abstract

The vast majority of goat skin processed by traditional tanneries comes from small rural producers. Thus, with the predominance of rustic creation, slaughter, and skinning methods, the batches of hides processed by tanneries have a very heterogeneous quality. Thus, there is a need to categorize the samples according to the quantity and location of defects. The categorization process is subjective and strongly influenced by the experience of the professional classifier, causing a lack of homogeneity in the composition of the goat hide lots for sale. Aiming to reduce failures in the categorization of goatskin samples, the authors investigate the application of computer vision and artificial intelligence on a set of previously categorized wet blue goatskin photographic samples. That said, is analyzed the capacity of different classifiers, with different paradigms, in detecting defects in goatskin samples and in categorizing these samples among seven possible quality levels. A hit rate of 95.9% was achieved in detecting defects and 93.3% in categorizing quality levels. The results suggest that the proposed methodology can be used as a decision aid tool in the qualification process of goat leather samples, which can reduce sample labeling errors.

## 1 Introduction

According to M. Gutterres [1], the production of leather has increased worldwide, at the same time as there has been a shift in the production base from developed countries to developing countries. Therefore, it is due to a combination of factors related to the availability of raw material, market, costs, among others.

Gomes Leal, Penna Rocha and Rocha Junior [2] explain that because it is easy to adapt to the semi-arid climate, the caprine species becomes an excellent option for livestock in the caatinga biome, which is widely found in Brazil and other tropical countries. Several parts of the animal can be used, with emphasis on the themes at, milk, meat and skin. In the case of leather, the concern with the management of the goat must be increased, as the skin is easily damaged when there is no care with the control of ectoparasites, places where the animal moves (damage when touching barbed wire fences and spines) and in the slaughter and removal of the skin for further processing.

As commented by Aslam et al. [3], in a changing global scenario, the manufacturing industry constantly demands higher product quality and higher productivity to satisfy customer requirements and reduce rejection costs. The increased demands for objectivity, reliability and efficiency have required the addition of automatic inspection systems in the traditional leather industry. Surface defects reduce the quality and value of the skins.

S. Liong et al. [4] cite that the defect inspection process is essential in the leather industry to control the quality of finished products. However, the inspection occurs visually, it is tedious, laborious, time-consuming, causes eye fatigue and, is often prone to human error.

## 2 Related works

Amorim, et al. [5] propose a work to classify flaws in goat leather samples in two types of images: raw leather and wet blue. For the attributes generation, Gray-Level Co-Occurrence Matrix (GLCM), Gabor filters and two color spaces are used. From the attributes obtained, 3 (three) experiments are carried out. The first one consists of classifying the raw data, without preprocessing them, using the classifiers K-Nearest Neighbors (KNN), Fischer classifier (based on Bayes rule) and Support Vector Machine (SVM), in which, it stands out the application of the KNN classifier, with an average accuracy rate of 95.9% for rawhide skins and 93.76% for wet blue. In the second experiment, attribute reduction techniques such as FisherFace, Chen Linear Discriminant Analysis (CLDA), Direct LDA (DLDA), Yang LDA (YLDA) and Kernel LDA (KLDA) are applied. Then, the resulting data is again classified with the same classifiers as the first experiment. In this step, the best combination appears through the CLDA and KNN, obtaining an average accuracy rate of 92.2% for raw leather parts and 90.3%, for wet blue. The third experiment consists of detecting the flaws, informing their location and comparing them with those reported by the specialist professional, in a visual analysis. As a result, the system found several false positives. The author of the work reports that one of the reasons for which such a problem should occur is the quality of the training set, as it does not have enough samples to discriminate all possible defects.

E.Q. Santos Filho, G. Barreto, and J. Daniel Santos [6] propose a model for classifying skin samples in the wet blue stage. The set of images is divided into 7 types of classes in which the author divided them into Upper Class (classes 1 to 5) and Lower Class (classes 6 and 7). The work performed consists of converting the obtained images (3264 x 2448 pixels) to gray levels and, later, resizing them to sizes of 40, 60, 80 and 100 pixels. Then, the characteristics are extracted using the Column-Variance (VAR), Haar wavelet transform (HAAR), Non-Negative Matrix Factorization (NMF), Principal Component Analysis (PCA) and GLCM. After the feature vectors are generated, the classification is made using the classifiers Least Squares (LS), Extreme Learning Machine (ELM), Regularized Linear Gaussian Classifier (RLGC) and SVM. In the work, there is also the elaboration of the rejection option in order to increase the reliability of the classifiers. The best results of the proposed work have an average accuracy rate of 85.56%, without the rejection option, through the VAR extractor and ELM classifier. With rejection option, 97.32%, with PCA and SVM.

Nogueira de Aquino [7] proposes the classification of goatskin samples using 3 (three) variations of the Local Binary Pattern (LBP) method: uniform LBP, rotation-invariant LBP and uniform and rotation-invariant LBP. For the classification, the KNN and SVM classifiers are used. After analyzing the classification from each extractor, it is also proposed to extract characteristics with multi-resolution, that is, the combination of 2 (two) or 3 (three) LBP operators. At the end of the work, it is noticed that the best average accuracy is 86.14%, obtained from the combination of 3 (three) LBP operators and SVM classifier.

Renato F. Pereira, Cláudio M. S. Medeiros, and Pedro P. R. Filho [8] propose defect detection and leather qualification. Initially, they fragment each goat leather into several squares windows with sizes of 51, 101, 151, 201, 251 and 301 pixels. Attribute extraction methods like Gray Level Co-occurrence Matrix (GLCM), Local Binary Patterns (LBP) and Structural Co-Occurrence Matrix (SCM) are applied in each window. Then, the defect classification is implemented by K-Nearest Neighbors (KNN), Multilayer Perceptron (MLP) and Support Vector Machine (SVM). Considering only defect classification, the best accuracy comes from the combination of 51 x 51 pixels windows, LBP extractor and MLP classifier, with an average accuracy of 90%. For qualification, the authors initially use the trained classifier to identify each defective window contained in the leather sample. Then, they identify the position of each defective window in the sample. Then, they use a mathematical algorithm to identify the convex areas free from flaws in the leather sample. Numerical attributes related to defect-free areas are generated and supplied to an SVM classifier, which obtains an accuracy of 86% in leather qualification, among the 7 possible categories.

Liong, S. et al. [4] propose a system for detecting and segmenting tick-bite-type defects in bovine leather samples. According to the authors, the proposal to work with this type of defect occurs because this problem is often overlooked by human inspection. The authors use a LED lamp as light source, a 24.2 megapixels camera (2400 x 1600 pixels spatial resolution) to acquire images, a robotic table 6-axis arm (model DRV70L from Delta Electronics, Inc.), and the Mask Region-based Convolutional Neural Network (Mask R-CNN) as a learning method. The methodology consists of inserting leather samples manually on a table in which the robotic arm moves in a linear way, from top to bottom, analyzing the pieces and, using the camera, digitizing the image to be analyzed. Thus, from the use of a computer, the detection of regions with this type of defect occurs, in which each identified problem contains its coordinates X and Y saved for later use. With the classification process completed, the robotic arm again passes through the entire piece of leather in order to, from a chalk inserted at its tip, highlight the regions previously detected as a defect. In the experiment 84 images are used for training and 500 images for tests, which obtain an accuracy of 70.35%.

## 3 Acquisition of sample sets

**Figure 1:**
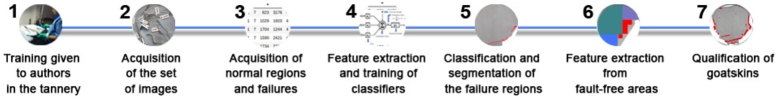
Methodological procedures carried out.

The methodology applied for the elaboration of the decision support system is represented in Fig. 1. In this way, as presented, the authors start the process by conducting training, carried out in a tannery, in order to obtain greater knowledge related to the detection of failures and definition of the quality level of the leather samples.

**Figure 2:**
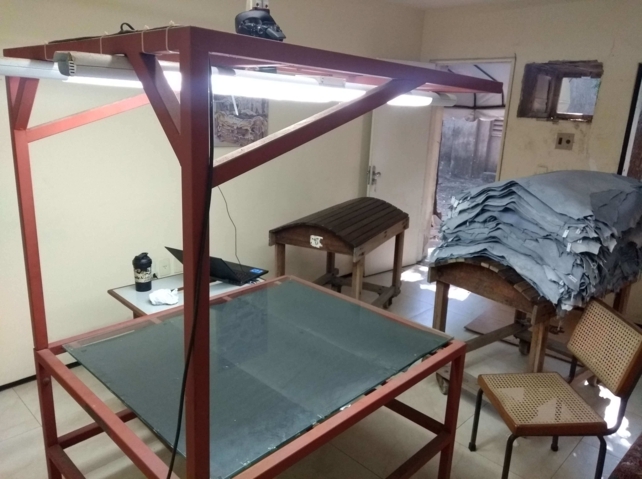
Table for digitizing leather samples.

The authors used the structure developed by Edmilson Q. S. Filho, José D. A. Santos and Guilherme A. Barreto [6] to acquire images of goat leather. The structure, illustrated in Fig. 2, has a table with a black background, in order to facilitate the extraction of the background of the object of interest in each image; own lighting, composed of two fluorescent lamps of 40 W; camera support installed 1.5 m above the table's surface; a photograph camera Canon Rebel T3 12.2 MP (55x optical zoom and EF-S 18-55mm lens); and a computer, using the Canon EOS Utility® software.

To make the study feasible, the authors used samples of goatskin available in the tannery stock, which were previously categorized by a very experienced expert, he has over 30 years of experience in this type of work, here called expert A. Were made 312 photographic records in the JPEG (Joint Photographics Experts Group) format.

These samples were coded, mixed, and subsequently recategorized by another specialist, also quite experienced, here denoted as specialist B. Table 1 presents the results of the categorization of both experts. In this table, the main diagonal indicates the number of samples that were equally categorized by the two experts. As can be seen, the coincidence percentage in the classification of the two experts is only 41.9%, which attests to task difficulty. This result is due to the fact that the process occurs by hand, and variables such as tiredness, inadequate lighting, among others, can lead to erroneous qualifications.

**Table 1:**
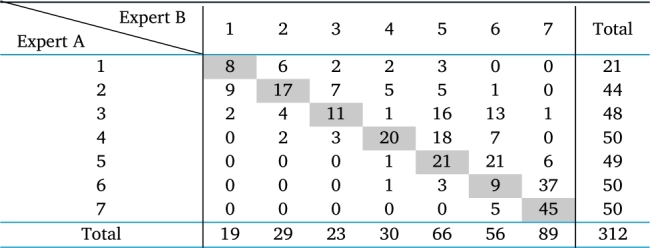
Confusion matrix in the labeling of wet blue leather samples by two experts.

It can be seen that, among the 312 samples, expert A categorized 21 samples as quality 1, while expert B categorized 19 samples. This difference is acceptable. However, for the lower quality samples, there are great divergences. As an example, expert A categorized 50 samples as quality 7 (lowest quality), while expert B categorized 89 samples, which attests to task difficulty. This result can be due to the fact that the process occurs by hand, and variables such as tiredness, inadequate lighting, among others, can lead to erroneous qualifications.

In this table, the main diagonal indicates the number of samples that were equally categorized by the two experts. As can be seen, the coincidence percentage in the categorization of the two experts is only 41.9%

There is a greater degree of caution on the part of Expert B, note that he has taken a slightly more pessimistic approach to the qualification given by Expert A.

While identical skill levels among experts may be considered low, this difficulty can be remedied at a later stage, called finishing. In it, the leather undergoes several processes such as cutting, painting, among others, which greatly reduces this inequality between them, as it greatly reduces the number of failures in the pieces.

It should also be noted that there is no standardized quantity of quality levels. The tannery can define its own standard based on the leathers purchased, where they will be applied or sold. For example, E.Q. Santos Filho, G. Barreto and J. Daniel Santos [6] consider only two types of quality: high, for good quality leather, and low, for bad leather. It is also possible to consider 5 types of quality: level 1, with only 10% defects, level 2, between 10 and 30%, level 3, between 30 and 50%, level 4, plus 50% defects, and leve 5, considered rejected or only 4 levels as excellent, good, regular and bad.

It is important to inform that despite the divisions presented, the fault detection process and definition of the quality level are based on the same methodologies presented in this work, that is, based on the number of faults and their locations.

From here, the authors form the dataset for the classifier design only with samples that had coincident categorization by the two experts. Thus, the degree of uncertainty in the sample labeling is lower, which can give the properly trained classifier greater reliability.

It should be noted that this article can be divided into two steps: the detection of defects and the qualification of wet-blue goat leather. The first one considers the acquisition of samples, the elaboration of data sets, and the use of classifiers for failure detection. For the second stage, it is considered the acquisition of data sets based on fault-free regions and the use of classifiers to obtain their quality level.

## 4 Defect detection in goat leather

**Figure 3:**
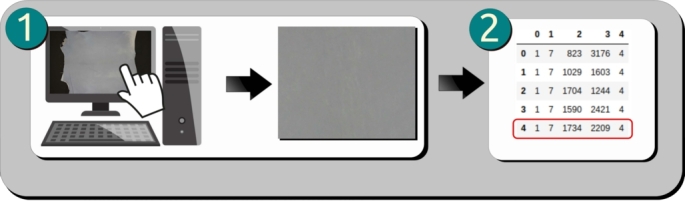
Procedure performed to prepare the defect report.

After acquiring the photographic records of the goat leather samples, the authors open the images in image editing software and, with the participation of professional classifiers, identify and locate each defect found. A file is generated containing the type of each defect, the identification of the image file in which the defect is found and the coordinates X and Y of the location of the defect in the image. This procedure is illustrated in Fig. 3.

Thus, 2250 defect images and 1669 defect-free leather images were cataloged. Table 2 illustrates the most common defects.

**Table 2:**
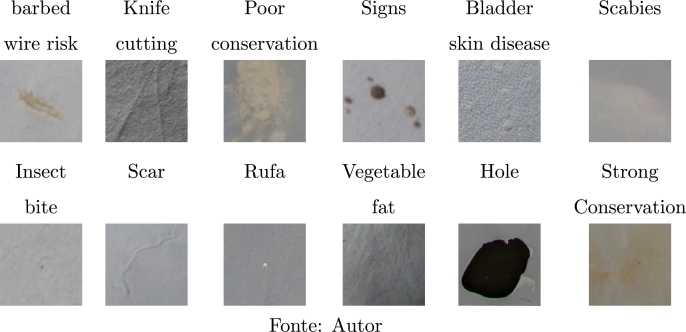
Main defects found in goat leather samples.

Then, the authors carried out an exhaustive investigation to define the window size, centered on the coordinates *X* and *Y*, most appropriate for the application of attribute extraction methods, seeking the best compromise between execution time and sample characterization.

For each window size, the authors evaluated the performances of several combinations of extracting attributes methods and classifiers based on three different paradigms.

Renato Pereira [9], in his master's work, also related to the detection of flaws in goatskin, used fifteen segmentation techniques (1- Rear Projection, 2 - Black Hat, 3- Canny, 4- Clustering, 5 - Convex Shell, 6- Find Contours, 7- Hough Transform for Circles, 8- Hough Transform for Lines, 9- Image Moments, 10- Hough Laplace Transform, 11- Region Growth, 12- Standard Deviation Filter, 13- Sobel, 14- Threshold of Otsu [10], and 15-Tophat) to detect failures in an automated format. However, these techniques allowed a maximum accuracy of only 50% in failures location. That way, the authors believe that for this problem, due to the heterogeneity of failures, manual work can be more robust, as it is carried out with the help of professionals of the tannery, who have years of experience in this type of work.

### 4.1 Attributes extraction

R.C. Gonzalez and R.E. Woods [11] explain that a computer vision system is composed of the acquisition, combination, feature extraction, and classification steps. In this way, the solutions are based on image processing and analysis techniques, as it allows extracting information from them.

The attribute extraction methods evaluated are the Local Binary Pattern - LBP, presented by T. Ojala, M. Pietikäinen, and T. Mäenpää [12] and the Gray Level Co-occurrence Matrix - GLCM, presented by R.M. Haralick, K. Shanmugam, and I. Dinstein [13]. It is noteworthy that these were chosen because they presented better results in the works cited in Section 2, thus, the authors used the literature to choose such methods.

D. Huang et al. [14] state that in recent years, LBP has sparked a growing interest in image processing and computer vision. This method efficiently summarizes local image structures, comparing each pixel with its pixels neighbors. P. Mohanaiah, P. Sathyanarayana, and L. GuruKumar [15] cite that one of the most important properties of this operator is computational simplicity.

W.R. Schwartz and H. Pedrini [16] present that the GLCM describes texture through a set of characteristics for the occurrences of each level of gray in the pixels of the image considering multiple directions. In this way, according to S. Annadurai and R. Shammugalakshmi [17], the size of the matrix is determined from the distinct number of levels of pixels contained in the original image. Haralick, K. Shanmugam and Dinstein [13] proposed a method which generates 14 statistical measures of texture. However, only 13 are actually used, since the latter presents computational instability.

**Figure 4:**
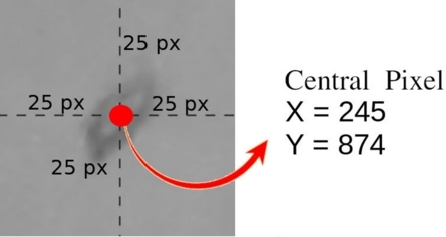
Example of fragment size used.

For each attribute extraction method, various window sizes were tested. In Fig. 4 one can see an example of a 51 pixels window.

Square windows are considered with *T* pixels on each side, each centered on the X and Y coordinates, which represent the center pixel of the regions saved in the report. The tested window sizes are 11, 21, 31, 41, 51, 75, and 101 pixels.

### 4.2 Classifiers and training/testing data sets

According to R. Kruse et al. [18], complex configurations of problems in different technical, commercial, and financial aspects evoke a growing need for computer applications that must show behavioral intelligence.

A. Burkov [19] explain that machine learning is a subfield of artificial intelligence that is concerned with building algorithms that, to be useful, rely on a collection of examples of some phenomenon. These examples can come from nature, human actions, or generated by another algorithm. Machine learning can also be defined as the process of solving a practical problem based on data sets. That said, in this paper, the authors used the following classification algorithms: Perceptron, Logistic Regression, K-Nearest Neighbors (KNN), Fisher's classifier, Gaussian Naïve Bayes, Multilayer Perceptron (MLP), and Support Vector Machine (SVM).

Perceptron is inserted in order to verify a possibility of linear separability between the classes of the data sets. It was implemented using learning rates with values of 0.01, 0.05, 0.1, and 0.5, with the possibility of linear or exponential reduction of them and signal activation function.

The Logistic Regression is a very simple algorithm, with a better possibility of converging in non-linear problems, it is implemented with penalty parameter C assuming values like 0.01, 0.1, 1.0 and 10.0.

From the data sets obtained during feature extractions, the authors noted clusters of samples, which makes it a good indicator for the use of one of the simplest alternatives for classification, the K-Nearest Neighbors (KNN). The classifier is implemented using Euclidean distance and K values equal to 3, 5, 7, 9, and 11.

Unlike KNN, which proves to be quite slow when using a large amount of data, the Fisher's classifier based on Bayes rule is a “probabilistic classifiers” and is easily adaptable to different data set sizes. Its parameterization and project process can be carried out in linear time. A simpler version of Fischer's classifier, which assumes strong independence between features, is the Gaussian Naïve Bayes. Both are investigated in this article.

For the MLP classifier, several parameters were evaluated: Initial learning rates are tested, ranging from 0.1 to 0.5; linear or exponential learning rate decay; hyperbolic tangent or logistic function as activation functions; and several numbers of hidden neurons, ranging 2 and 100 neurons.

Regarding to SVM classifier, 4 types of kernel are used: linear, RBF, polynomial and sigmoid. The value of the penalty parameter C is evaluated from the following values 0.01, 0.1, 1.0 and 10.

For the Logistic Regression algorithm, the training process stops when achieves 2000 iterations and/or the error rate achieves 10^−5^.

The authors used 1335 samples from the “normal” class and 1335 samples from the “defect” class to compose the training set. The samples were randomly selected from 1669 and 2250 samples, respectively from the “normal” and “defect” sets. The authors believe that this balance in the number of samples can avoid trends in the binary classification. The rest of the data, that is, 334 samples of the “normal” class and 915 samples of the “defect” class, make up the test set.

For all classifiers the K-Fold cross-validation technique is used, with a value for K equal 10. The application of this method consists of dividing the total set of training data into K subsets of the same size. Thus, each subset is used for tests, while the rest of the set is applied to estimate the parameters.

### 4.3 Results for defect classification

**Figure 5:**
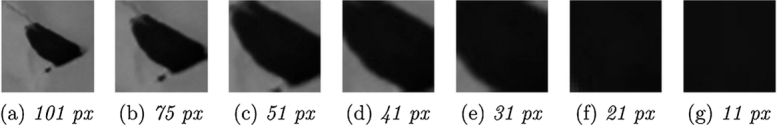
Examples of fragment sizes used in a wet blue goatskin sample.

During the defect detection process, the authors realized that by setting very small fragment size, several classification errors were obtained. As shown in Fig. 5, different fragment sizes represent different forms of the problem. Note, that the image on the left has a large number of gray tones, and these are reduced as the fragment is reduced.

The best results were achieved with the data set formed by applying the GLCM attribute extraction method to windowed images with 51 pixels.

The average results and the standard deviation for the hit rates in 50 runs of the three best results obtained can be seen in Table 3.

**Table 3:** Best averaged results obtained in the defect identification process.

Classifier	Acc (%) / Std. (*σ*)	
	training	test
Logistic Regression	94.75 ± 0.32	93.63 ± 0.56
MLP	94.95 ± 1.03	95.05 ± 1.67
SVM${}_{RBF}$	96.59 ± 0.17	95.95 ± 0.38

Referring to the two best results in test set, the configuration of the MLP networks is as follows: Initial learning rates of the hidden and output layers of 0.5 and 0.3, respectively; linear decay in learning rates; 65 hidden neurons; logistic activation function; and rejection range of 0.1.

In the case of SVM, the best kernel was RBF. The mean of support vectors is 368 and parameter C equals 10. With this configuration it was possible to obtain the best model for fault detection, its result is shown in Table 4.

**Table 4:**
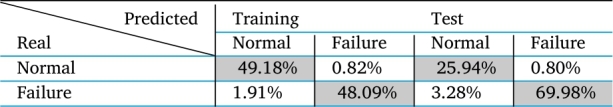
Confusion matrix of the best SVM model obtained for fault detection.

When analyzing Table 4, it is possible to notice a low number of incorrect classifications. These errors occur most frequently when identifying samples with defects.

**Figure 6:**
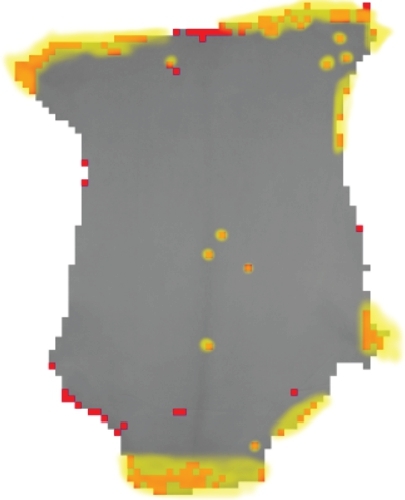
Comparison between a visual inspection by an expert (in yellow) and the proposed algorithm (red).

In Fig. 6, the authors present the result of applying the best SVM classifier in the defect identification in a sample of goat leather. Image fragments marked in red were identified as defects. For comparison purposes, a tannery expert identified the regions marked in yellow as defect.

From here, defect detection will be performed only by a Support Vector Machine, on attributes generated by GLCM on image fragments of 51 pixels.

## 5 Goat leather qualification

For the goat leather qualification, firstly it is necessary to perform a preprocessing on the sample image, performing procedures for filtering and removing the background. Then, the leather image is fragmented into several 51 pixels windows and a matrix is constructed, in which each element represents a specific window. Each element of the matrix is assigned the value “0”.

Each window is subjected to the defect detection process presented in Section 4, in which an SVM classifier assigns the value “0” to the window classified as belonging to the class “defect” and the value “1” to the window classified as belonging to the “normal “class.

**Figure 7:**
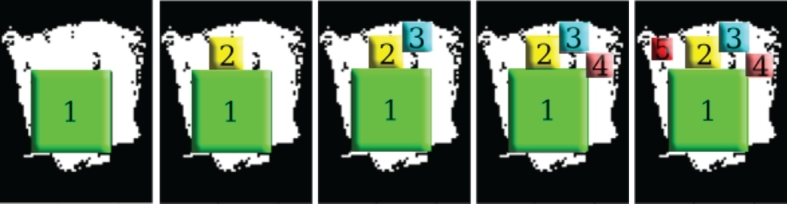
Detection of flawless regions.

Then an algorithm is applied to find the defect-free convex regions in the matrix. The detection of these defect-free areas is based on a dynamic programming problem explained by Dhaval Dave [20]. The method is applied recursively until find the largest 5 defect-free convex regions, as is suggested in Fig. 7.

Seven attributes are generated based on defect-free areas. These attributes are listed in Table 5.

**Table 5:** Attributes extracted from the process of detecting defectless regions.

Attribute	Description
1	Total defect-free area;
2	Total area calculated over 5 greatest defect-free convex regions detected;
3	Area of the largest defect-free convex region detected;
4	Percentage of the largest defect-free convex region detected regarding only the total of defect-free areas;
5	Area of the smallest defect-free convex region detected regarding only the total of defect-free areas;
6	Percentage of the smallest defect-free convex region detected regarding only the total of defect-free areas;
7	Standard deviation calculated over the defect-free areas detected.

From here, each goat leather sample image is characterized by the 7 attributes defined in the Table 5.

### 5.1 Goat leather sample qualifier project

In this research, only the leather samples that obtained the same categorization by the two experts are considered to compose the data set. The data set was composed according to the undersampling technique. Thus, considering the class with the smallest number of samples (quality 1 - 8 samples), 75% of them were used to compose the training set. The other classes contribute an equal number of samples to the composition of a balanced set with a total of 42 samples. The test data set is composed by the rest of samples, that is 89 samples.

The authors use the follow classifiers to perform the qualification task: Perceptron, Logistic Regression, K-Nearest Neighbors (KNN), Fisher's classifier, Gaussian Naïve Bayes, Multilayer Perceptron (MLP) and Support Vector Machine (SVM). However, the authors present in Table 6 only the average results of the three best classifiers.

**Table 6:** Averaged results (50 executions) obtained in the goatskin qualification process.

Classifier	Acc (%) / Std. (*σ*)	
	training	test
Gaussian Naïve Bayes	95.79 ± 0.97	89.56 ± 2.35
MLP	98.0 ± 2.64	74.14 ± 2.97
SVM${}_{RBF}$	95.02 ± 11.99	81.27 ± 4.59

The three classifiers have average hit rates above 95% in the training set. However, the Gaussian Naïve Bayes classifier stands out from the other two by achieving an average hit rate in test set of 89.56%. Table 7 presents the confusion matrices in the training and test sets of the best Gaussian Naïve Bayes classifier among 50 achievements.

**Table 7:**
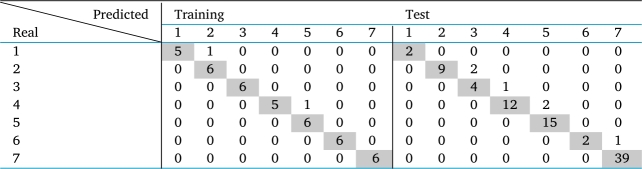
Best result in qualifying goat leather samples using the Gaussian Naïve Bayes classifier.

As shown in Table 7, there are few classification errors. These, when they occur, are identified with classes close to the real ones, making it an acceptable qualification error for the tanneries. The referred classifier presents hit rates in the training and test sets of 95.2% and 93.3%, respectively.

In Table 8 one can see the images of seven samples correctly categorized by the Gaussian Naïve Bayes classifier, one for each quality level. The red marks are the 51-pixel square image fragments detected as having defects.

**Table 8:**
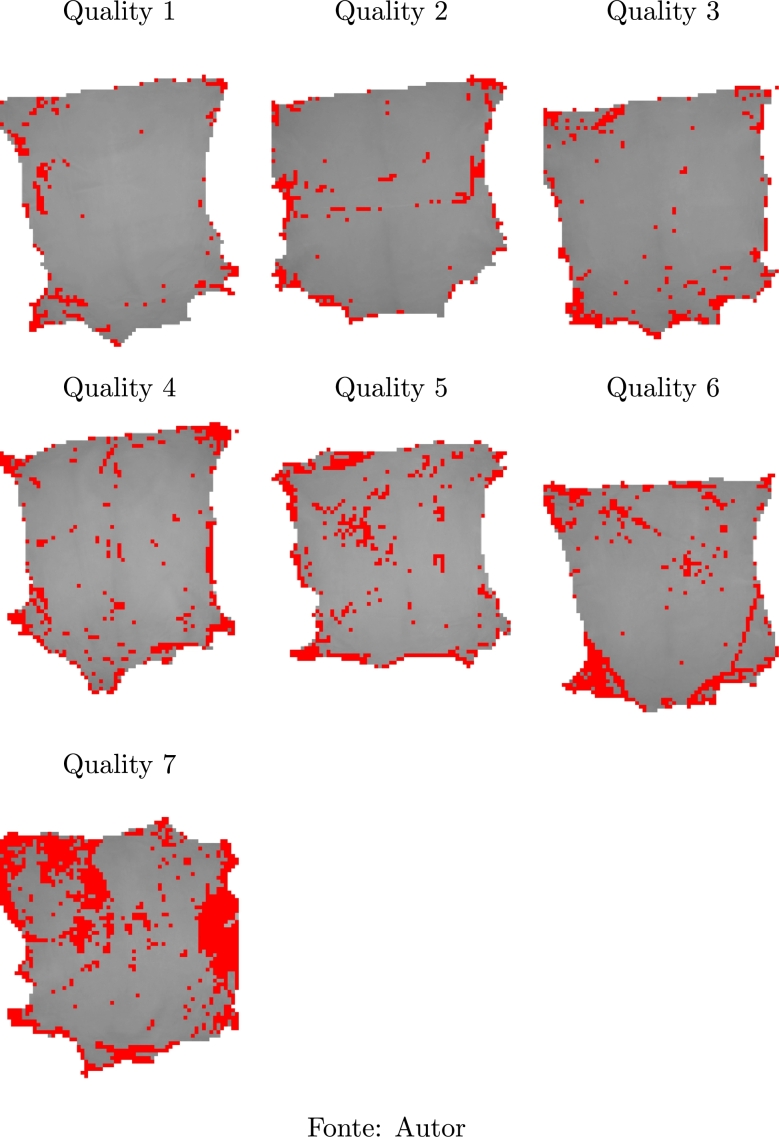
Main defects found in goat leather samples.

It is noticed that the samples with the best qualities are those with few defects in the central region. This is in accordance with the qualification methodology employed by the experts, as the number of defects found in the sample may not be as relevant in determining quality if they are located at the edges of the sample.

### 5.2 Comparison between classifier and experts

As commented in Section 3, the coincidence percentage in the classification of the two experts is only 41.9%. Now, the authors assess the coincidence percentages between the labeling by the classifier and each of the experts. In Table 9, Table 10 one can see the confusion matrices between the classifier and experts A and B, respectively.

**Table 9:**
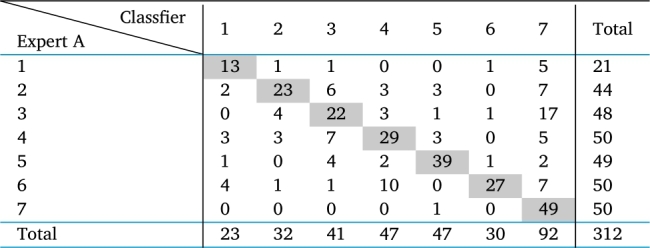
Confusion matrix between classifier and expert A.

**Table 10:**
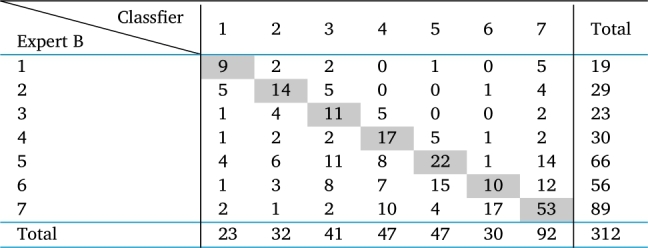
Confusion matrix between classifier and expert B.

The coincidence percentage in the classification of the classifier and expert A is 64.74%, while between the classifier and expert B it is only 43.59%. It is important to point out that in this analysis we are also considering all the samples used for training and testing the classifier.

It was expected that there would be a greater coincidence between the classifier and the expert A, as he is more experienced and is the instructor in the internal training programs in the tannery. It is noticed that only for qualities 1 and 6 do experts qualify similar numbers of samples. For all others there is great disparity. Expert B seems to be more rigorous, as it tends to qualify more samples in the lower quality categories.

## 6 Conclusions

As seen, there is a great difficulty in qualifying wet blue leather pieces, as currently a very artisanal process is used, which provides several wrong qualifications. It was found that one of the greatest difficulties of this research was to obtain a reliable set of images since the identical qualification level between two specialists was only 41.9%. Thus, to guarantee this reliability, only leather pieces with the same quality level defined by the two specialists were used for the machine learning process, which reduced the number of samples from 312 to just 131.

The qualification process consists of two steps: the detection of defects and, from there, the qualification of wet blue goatskin. Although simpler, it is essential that the first step is well executed so that the entire process takes place satisfactorily. In this way, the occurrence of incorrect fault detection can lead to incorrect qualification of goat skins. Thus, it can be said that the second stage depends entirely on the results obtained in the first.

To carry out the first step, that is, for the detection of failures, seven sets of images were prepared, from windows of 11, 21, 31, 41, 51, 75, and 101 pixels, each composed of 1669 samples of classes without failures and 2250 with defects. Attribute extraction was performed using the LBP and GLCM methods and, for the process of classification of failure or non-failure, Perceptron, Logistic Regression, KNN, Fisher classifier, Gaussian Naïve Bayes, MLP, SVM were used. In this first step, the best result was obtained from the combination of the image window size of 51 pixels, GLCM extractor, and SVM classifier${}_{RBF}$, obtaining 95.9% correctness.

The second stage, that is, the qualification of the leather pieces, is based on the detection of defects. In this, the largest fault-free regions are considered, and from these, seven attributes are acquired for the formation of the dataset. To define the quality level, the same machine learning algorithms mentioned above are used. As the best result, an accuracy rate was obtained, from the Gaussian Naïve Bayes classifier, of 93.3%.

As presented in this work, an index of 93.3% of correctness was observed when the wet blue leather pieces that obtained the same quality level by the two specialists are classified. However, there is a reduction in the success rate when a classification is made based on the distinction of the quality level given by each one of the professionals. It is noteworthy that these results were expected, given the great difference between the qualifications they attributed.

Finally, it is believed that the proposed system becomes an effective instrument to be used as a decision support system, as it is built from a set of images that obtained the same level of quality by two specialists, and from this, was obtained an accuracy of 93.3%, which makes it suitable for application in this type of industry.

## Declarations

### Author contribution statement

C.E.B. Sousa: Conceived and designed the experiments; Performed the experiments; Analyzed and interpreted the data; Contributed reagents, materials, analysis tools or data; Wrote the paper.

C.M.S. Medeiros: Conceived and designed the experiments; Analyzed and interpreted the data; Contributed reagents, materials, analysis tools or data; Wrote the paper.

R.F. Pereira: Conceived and designed the experiments.

A.A. Neto, M.A.V. Neto: Contributed reagents, materials, analysis tools or data.

### Funding statement

This research did not receive any specific grant from funding agencies in the public, commercial, or not-for-profit sectors.

### Data availability statement

Data will be made available on request.

### Declaration of interests statement

The authors declare no conflict of interest.

### Additional information

No additional information is available for this paper.
